# Supplementary material for: rtmsEcho: An Open-Source R Package for Automated Analysis of Acoustic Ejection Mass Spectrometry Data
Source: Anal Chem. 2025 Sep 12;97(37):20444–52. doi: 10.1021/acs.analchem.5c03730 (PMC12462762; doi:10.1021/acs.analchem.5c03730)
Supplement: Supplementary file 1 [file ac5c03730_si_001.pdf]

Supporting Information

rtmsEcho: An Open-Source R Package for Automated Analysis of Acoustic Ejection Mass Spectrometry Data

Mary Ashley Rimmer[1†], Nathaniel Twarog[2†], Tharindu A. Ranathunge[1,3], Jingheng Wang[4], Yong Li[1], Taosheng Chen[4], Anang A. Shelat[2], Lei Yang[1*]

[1] Analytical Technologies Center, Department of Chemical Biology and Therapeutics, St. Jude Children's Research Hospital, Memphis, Tennessee

[2] Lead Discovery Informatics, Department of Chemical Biology and Therapeutics, St. Jude Children's Research Hospital, Memphis, Tennessee

[3] Compound Management, Department of Chemical Biology and Therapeutics, St. Jude Children's Research Hospital, Memphis, Tennessee

[4]Department of Chemical Biology and Therapeutics, St. Jude Children's Research Hospital, Memphis, Tennessee

[†]Co-first authors

*Corresponding author; e-mail: lei.yang@stjude.org

Table of Contents

Description	Page No.
**Figure S1.** Distribution of the molecular weights of compounds analyzed by AEMS and rtmsEcho	S3
**Figure S2.** Comparison of date readout from rtmsEcho and Sciex OS for QC plates	S4
**Figure S3.** Comparison of data processed by rtmsEcho and Sciex OS for a CYP3A4/5-selective assay	S5
**Figure S4.** Bland-Altman plot comparing results from SciexOS and rtmsEcho	S6

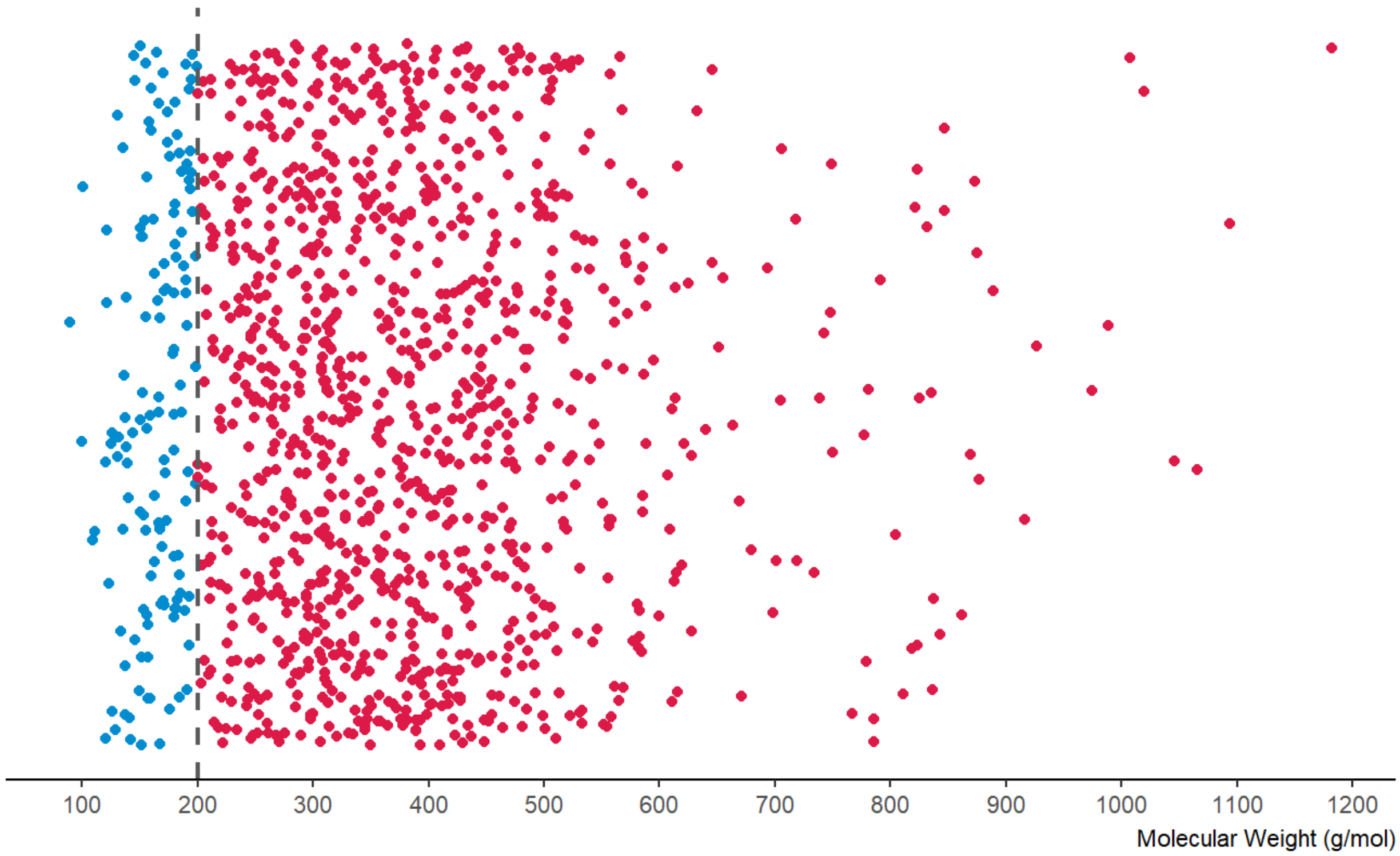

Figure S1. Distribution of the molecular weights of compounds analyzed by AEMS and rtmsEcho. Among the screening library, 146 compounds—representing nearly 10%—have molecular weights below 200 g/mol.

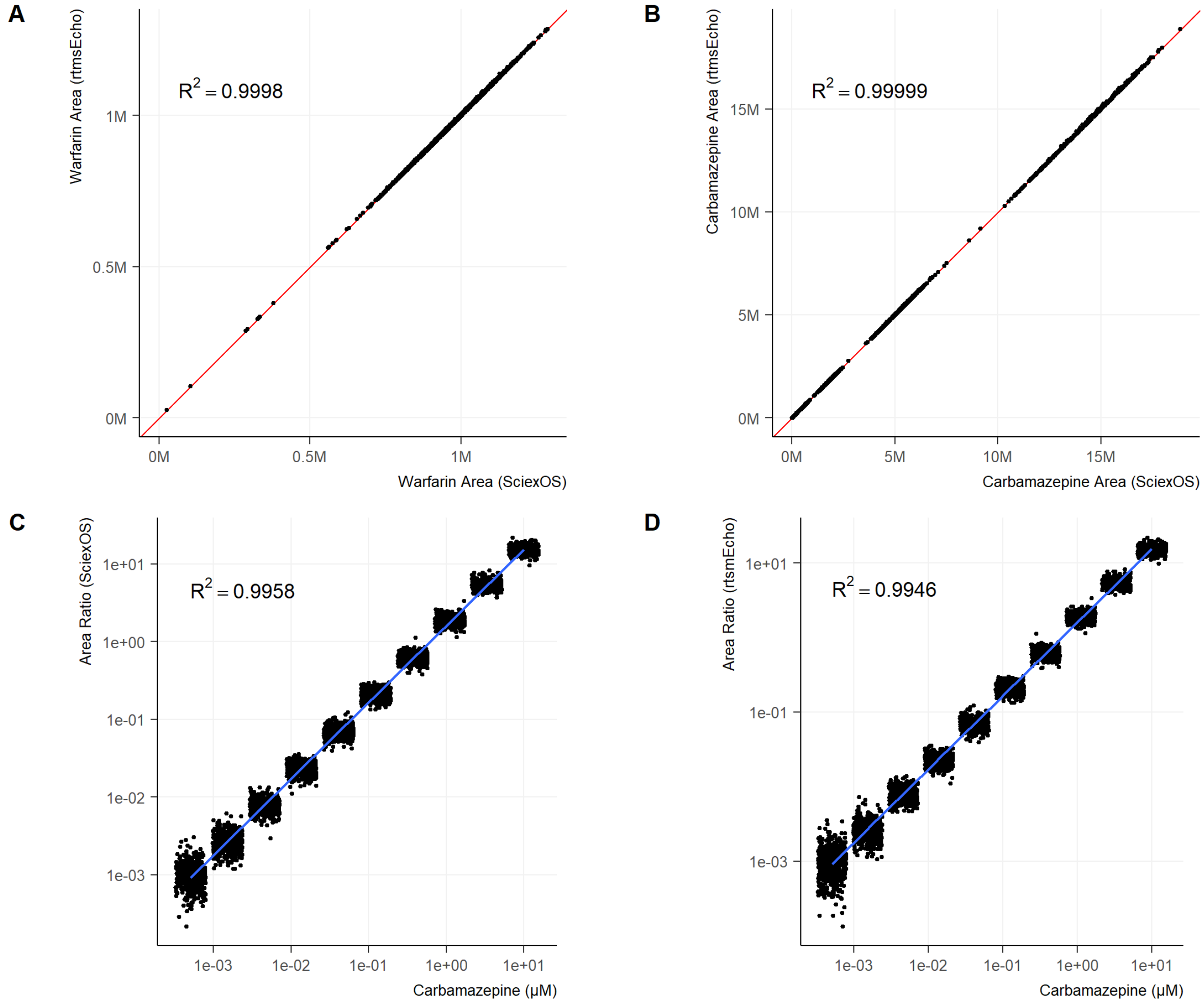

Figure S2. Comparison of date readout from rtmsEcho and Sciex OS using 20 Compound Management QC plates analyzed with MRM scanning mode. A, B) For raw quantification, Sciex OS and rtmsEcho produce nearly identical results for the compounds warfarin and carbamazepine. The estimated peak areas for both compounds show a strong correlation between methods with an R-squared value virtually indistinguishable from 1. C, D) When comparing the area ratios for carbamazepine estimated by Sciex OS and rtmsEcho to the actual carbamazepine concentrations on a logarithmic scale, both methods demonstrate nearly identical precision in their correlation.

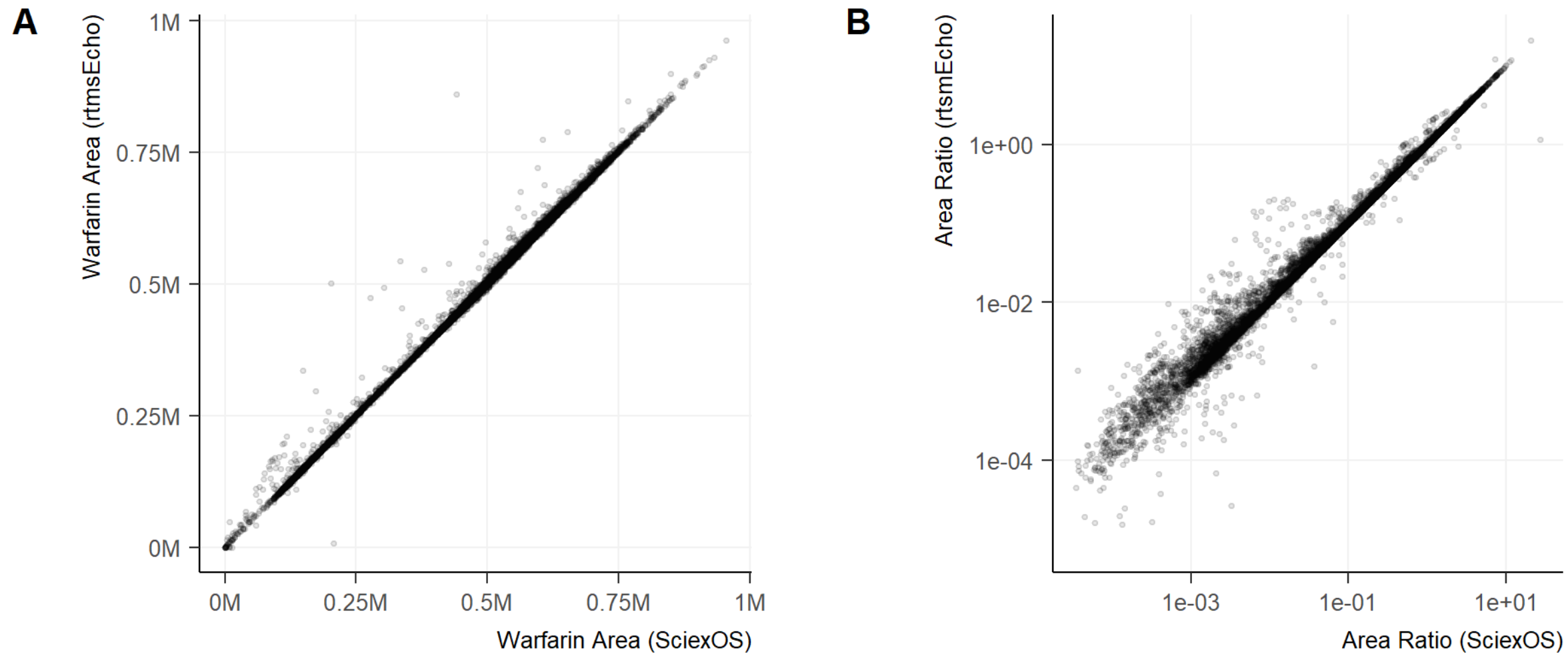

Figure S3. Comparison of data processed by rtmsEcho and Sciex OS for a CYP3A4/5-selective assay. A CYP 3A4/5 selective assay using MRM scan for nearly 1500 compounds shows that the quantification of area ratios by SCIEX OS and rtmsEcho remain closely aligned. Areas of the internal standard warfarin correlated on a linear scale with R-squared values of 0.9974 (A); area ratios for all tested compounds correlated on a logarithmic scale with an R-squared of 0.9895 (B).

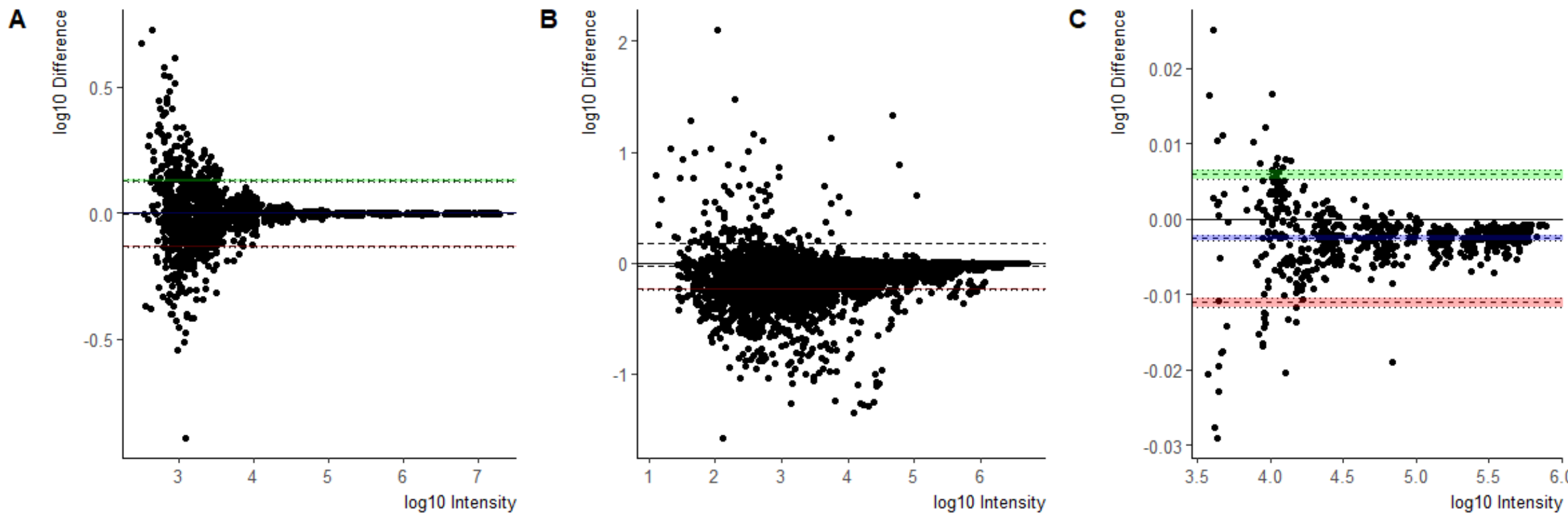

Figure S4. Bland-Altman comparing results from Sciex OS and rtmsEcho. Differences between Sciex OS analysis and rtmsEcho analysis of three sets of MRM scanning mode data was plotted using the Bland-Altman method. Larger errors at lower intensities reflect the reduced signal to noise ratio in the data when detection of analytes is low, but the average difference between the methods is highly consistent across all measured intensities. Furthermore, the error is indistinguishable from zero for calculated area. A) Carbamazepine areas from 20 Compound Management QC plates. B) Data from the CYP3A4/5-selective assay. C) Eight compounds used for Full Scan mode testing were run in MRM scanning mode for method comparison.
